# The development of an explanatory model for voluntary medical male circumcision in KwaZulu-Natal, South Africa

**DOI:** 10.4102/safp.v63i1.5346

**Published:** 2021-11-16

**Authors:** Celenkosini T. Nxumalo, Gugu G. Mchunu

**Affiliations:** 1 College of Health Sciences, School of Nursing and Public Health, University of KwaZulu-Natal, South Africa; 2 Faculty of Health Sciences, Durban University of Technology, Durban, South Africa

**Keywords:** explanatory model, medical male circumcision, primary health care, voluntary medical male circumcision, HIV prevention, HIV/AIDS

## Abstract

**Background:**

KwaZulu-Natal (KZN) remains the epicentre of the human immunodeficiency virus/acquired immunodeficiency syndrome (HIV/AIDS) epidemic in South Africa. The incidence of HIV infection in KZN necessitates cost-effective strategies to curb the spread of infection. Voluntary medical male circumcision (VMMC) has been adopted as an additional biomedical preventive strategy since 2010 in line with recommendations from the World Health Organization. Despite several attempts to scale-up VMMC to reach age specific targets to achieve immediate aversion of infections, the uptake of VMMC remains sub-optimal, particularly in KZN. The purpose of this study is to describe the processes that were followed in developing, describing and evaluating an explanatory model for VMMC in KZN, South Africa.

**Methods:**

A qualitative theory-generative phenomenographic study design was used to analyse the qualitative differences in primary healthcare stakeholders’ experiences, understanding and conceptions of VMMC in KZN, South Africa. The emerging results informed the development of the VMMC explanatory model for KZN, South Africa. The model development process followed four steps, namely (1) concept analysis, (2) construction of relational statements, (3) model description and (4) model evaluation. The criteria of relevance for the target audience – applicability, clarity, user friendliness and originality of work – were used to evaluate the model.

**Results:**

The model’s central premise is that the decision to undergo VMMC is shaped by a complex interplay of factors in the context or external environment of males (the extrinsic variable), which influences specific experiences, conceptions and understanding regarding VMMC (the influential/intrinsic variables). These collectively determine men’s responses to VMMC (the outcome variable).

**Conclusion:**

The model describes the process by which contextual, extrinsic and intrinsic variables interact to determine an individual male’s response to VMMC, thus providing a guide to primary healthcare providers on care, practice and policy interventions to support the uptake of VMMC in the rural primary healthcare context of KZN, South Africa.

## Introduction

The burden of human immunodeficiency virus (HIV) infection in South Africa, particularly in KwaZulu-Natal (KZN), is a major public health concern.^1,2^ Epidemiological data reveal that there are currently 7.5 million people living with HIV/acquired immunodeficiency syndrome (AIDS) in South Africa.^3^ It is estimated that the number of new HIV infections in South Africa is 200 000 per year.^3,4^

By the end of 2019, there were 72 000 AIDS-related deaths in the general population. The KZN province presently has more than 1 million people living with HIV/AIDS and is considered to be the epicentre of the country’s HIV/AIDS epidemic. The challenge of rising incidence and prevalence of HIV related infections, diseases and deaths have multifaceted complications affecting individuals, families, communities and society in general.^5^ In the 2019 and 2020 financial year, the state spent 20 billion rand on healthcare as a result of the burden of HIV/AIDS in South Africa.^6^

The escalating HIV/AIDS incidence and prevalence rate in South Africa, especially in KZN, necessitates a combination of curative and preventive strategies not only to halt the spread of the virus but also to prevent the future occurrence of infections.^7^ Voluntary medical male circumcision (VMMC) is one of the preventive strategies that have been adopted in KZN, South Africa, following recommendations by the World Health Organization (WHO).^8^ These recommendations were made following the results of three randomised control trials that demonstrated that VMMC offered partial protection against heterosexual transmission of HIV infection by up to 60%.^9^

Despite much efforts, and the increase in the number of males circumcised over the years, the statistical performance of VMMC continues to be below the stipulated age-specific targets in South Africa, particularly in KZN.^10,11,12^ The review of VMMC data over the years has indicated that since the introduction of VMMC in South Africa, just over 3 million VMMCs have been performed, with nearly a million still outstanding when compared with national targets. Until recently, in KZN, just over 1 million VMMCs have been performed since its reintroduction in 2010. Although concerted efforts have been made towards increasing the uptake of VMMC through a wide range of communication strategies, uptake remains low, particularly in the age group of 15–49-year-old males. A recent statistical report by the KZN Department of Health^13^ has revealed that almost 80% of the VMMCs performed are usually in the age group of 10–14 years. This means that only 20% of males above 15 years old have been circumcised medically. Although medical circumcision of the males in the age group of 10–14 years is also beneficial in the long term, circumcising males in the age group of 15 years and older are more significant as this age group is more sexually active; hence medical circumcision could result in the immediate aversion of HIV infection.^14,15^ Data on the efficacy of traditional male circumcision (TMC) reveals that whilst TMC offers some degree of partial protection against HIV infection, it is significantly lower when compared with medical circumcision.^16,17^ This is because the TMC does not often remove all of the foreskins, thereby allowing continued access to resident immune cells that are viral entry points for HIV infection.^17^

Research has shown that interpersonal factors, the social context, culture and religion all play a significant role in influencing the uptake of VMMC by males.^18,19,20^ From a socio-cultural perspective, the difference in cultural opinions between VMMC and TMC has been shown to be barriers to the acceptability of medical circumcision.^21,22,23,24^ This is most prominent in communities that practice TMC as a rite of passage into manhood.^25,26^ It is postulated that individuals often use tradition as a model to construct their lives from a biological and social standpoint. Therefore, improving uptake of VMMC in the context of such cultural differences is reliant on respect and engagement with indigenous practices related to TMC whilst simultaneously making concerted efforts to expose the scientific benefits of VMMC.

Similarly other studies conducted on the acceptability of medical circumcision have shown that healthcare workers (HCWs) and female partners are major influencers in terms of a man’s decision to undergo VMMC.^27,28^ Studies exploring female partners’ role in the uptake of VMMC have revealed their view of a circumcised man being more aesthetically appealing and masculine were motivators for men to undergo medical circumcision.^29,30^ From a healthcare perspective, it is postulated that the main barrier to the successful scale-up of VMMC in the priority countries is demand creation, so an understanding of the contextual barriers and facilitators for VMMC is crucial for demand creation.^31,32,33^

Current research on the contextual factors influencing VMMC has largely focused on males.^18,34,35,36^ There is a dearth of research on the roles of female partners and HCWs, who are also major influencers of uptake. Phili^37^concurs that there appears to be a paucity of literature regarding VMMC acceptance, attitudes and perceptions amongst HCWs, particularly in settings where VMMC has not historically been practised, such as in KZN. Contextual studies on demand creation for VMMC have neglected the cultural component of VMMC, which largely influences circumcision in KZN, South Africa.

Existing strategies to scale-up VMMC have mainly been based on the WHO joint strategic action framework,^38^ which is very broad in scope and may therefore not be specific to the KZN healthcare context. A recent model by Maibvise and Mavundla^39^ provided a framework for healthcare providers to promote uptake of VMMC in high HIV and low male circumcision prevalence settings. Whilst this model is instrumental in directing interventions to create demand for VMMC, the main assumptions of the model are based on a concept analysis rooted in the paradigmatic perspectives of Neuman’s Systems Model^40^ and the Health Belief Model.^41^ The context of the application of this model is also general and may not necessarily take into consideration the unique context of KZN especially from a rural primary healthcare perspective.

The current South African national guidelines on VMMC (2016) provided extensive theoretical knowledge and direction to all stakeholders on the provision of safe, effective and quality VMMC based on the underlying principles of ethical and quality healthcare service delivery. The guidelines also provide insight on the means of providing a cost-effective, efficient and effective service, which is aligned to current HIV policies and strategic plans so that the benefits of the service can be experienced.

The contents of the guidelines, however, are limited to clinical aspects of VMMC and as a result do not provide much guidance on how healthcare providers can contribute to ensuring uptake of the service, particularly at a primary healthcare level. The recent national Department of Health demand creation toolkit for VMMC^42^ provided some guidance, but it is based on a single behavioural theory and does not take cognisance of the cultural component of VMMC. It is also mainly directed towards uncircumcised males, omitting their influencers. The purpose of the present study was to develop and describe an explanatory model of VMMC in KZN, with specific emphasis on the rural primary healthcare context and with the aim of directing interventions of primary healthcare providers in supporting the uptake of VMMC in this context.

## Methodology

### Design

The researcher used a theory-generative, qualitative phenomenographic study design. The analytical qualitative aspect of the design that used an in-depth semi-structured interview permitted a holistic enquiry regarding VMMC in terms of implementation and uptake. This also included a focus on the qualitatively different ways in which primary health stakeholders’ experience, understand and conceptualise VMMC in a rural context influenced by religious and cultural norms. The use of a phenomenographic study design allowed for an analysis of qualitative differences in participants’ experiences, understanding and conceptions regarding the phenomenon. In so doing, a broad and holistic perspective regarding VMMC was explored. This informed the development of the explanatory model of VMMC in KZN, South Africa.

### Study setting

The study was conducted at six different primary healthcare facilities that offer VMMC services in the KZN province of South Africa. The selected health facilities serve a catchment population of approximately 110 000–250 000 males aged 15–49 years. Each health facility performs between 700 and 2500 VMMC’s on men and boys annually. The majority of VMMC’s are performed on boys aged 10–14 years. The data collection sites were predominantly rural settings, with participants who were mostly IsiZulu speaking. Male participants in this study harboured religious and cultural norms, which were found to be potential barriers to uptake of VMMC. The conception of VMMC as being unnatural, unnecessary and sinful were some of the competing norms found in study, which informed the development of the present model.^28^

### Sampling and recruitment

Purposive sampling was used to select the health facilities and participants for data collection. For the purpose of this study, primary healthcare stakeholders, namely healthcare providers, policymakers, circumcised males, uncircumcised males and female partners of both circumcised and uncircumcised males, took part in the study. Specific inclusion and exclusion criteria were used to recruit each of the different population groups; these are observed in the different studies conducted. A total of 85 participants took part in the study.

### Data collection

**FIGURE 1:**
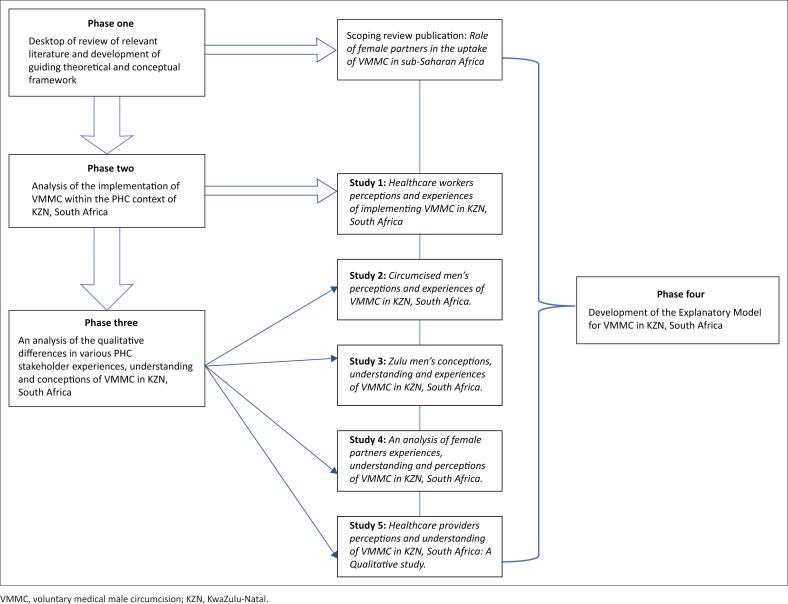
Outline of research studies and data generation process informing development of the explanatory model.

Five qualitative studies were conducted on the different population groups of the study in order to address the objectives of the study and ultimately the broad aim of the study, which was to develop an explanatory model of VMMC in KZN, South Africa – Figure 1 provides an outline of the research studies and data collection process, which collectively informed the development of the explanatory model of VMMC in KZN, South Africa. In each of the studies conducted to meet the individual objectives of the study, individual in-depth face-to-face interviews were conducted with the selected sample of participants from the various population groups of the study. A semi-structured interview guide was used to guide all interviews. The interview guide contained a demographics section and specific questions to elicit conceptions, understanding and experiences of VMMC in KZN, South Africa. This was done purely through audio recordings. The interview guide was written in English and Isizulu, and both languages were used during data collection. The researcher who conducted the interviews was bilingual; however, the assistance of a language specialist was sought to translate the data collection tools prior to data collection. The translator was also used to transcribe and translate data that were collected in IsiZulu. The researcher thereafter conducted the data analysis.

### Data analysis

Following data collection, the recorded data were transcribed verbatim and then analysed using phenomenographic data analysis procedures. In cases where the data were collected in Isizulu, the assistance of a language specialist was sought to transcribe the data in the original language and then translated into English before analysis. An independent verification of the translated data was performed with another language specialist in order to ensure that the meaning of the data was not lost. The data analysis was an iterative process, which followed a step wise approach as recommended by Sjöström and Dahlgren.^43^ Initially, the interview transcripts were read several times whilst listening to the audiotapes to ensure that data were accurately transcribed and to gain an overall understanding of the data. The second step entailed a more focused reading to extract similarities and differences from the data. Thirdly, significant aspects of the transcript were extracted. The fourth step involved a preliminary grouping of similar responses leading to the creation of an initial list of descriptive categories that were later refined through constant comparison with the transcript. The final set of categories was named, and the outcome space was formulated based on the internal relations between the categories of description.

### Trustworthiness

The criteria for ensuring trustworthiness were applied during data collection and analysis in all phases of the research study. The explanatory model was developed based on the findings of the studies conducted in this research project.

### Development of the explanatory model of voluntary medical male circumcision in KwaZulu-Natal, South Africa

The researcher followed the steps of theory generation as proposed by Chinn and Kramer^44^ in the development of the model. These steps are as follows: concepts analysis, construction of relational statements and model description. The resultant model that was developed in this study is described according to the following aspects: (1) purpose of the model, (2) assumptions of the model, (3) the context, (4) definition of concepts, (5) relational statements, (6) diagrammatic representation and description of the model. The description provided here details the steps that were taken in the process of developing the resultant model.

#### Step one: Concept analysis

To facilitate concept analysis, the three main components of concept analysis as outlined by Walker and Avant,^45^ which include (1) *concept identification*, (2) *concept definition* and (3) *concept clarification*, were followed.

**Concept identification:** The central concepts were derived from phases one and two of this study. These involved an analysis of the implementation of VMMC within the primary healthcare (PHC) context and the analysis of qualitative differences in PHC stakeholders’ experiences, understanding and conceptions of VMMC. Both phases of the study entailed the collection of primary qualitative data through individual in-depth face-to-face interviews. Phenomenographic data analysis processes were followed when data were analysed.

**Concept definition:** The key concepts were defined on the basis of an integrative review of relevant literature, theoretical underpinnings of the study and emerging findings. See Table 1 for definition of key concepts identified in the proposed explanatory model.

**TABLE 1:** Definition of key concepts in the model.

Concept	Definition
Influential (intrinsic) variables	Refers to those factors that influence receptiveness or acceptability of VMMC by males and their primary influencers (these are the varying experiences, understanding and conceptions).
Context (extrinsic variable)	Refers to the context in which influential factors and associated variables occur and interact to influence male clients’ responses to VMMC.
Outcome/response to VMMC	Refers to male clients’ uptake of VMMC based on an interaction of the influential variables. The outcome or response may result in improved uptake or a decrease in uptake of VMMC.
Enablers	Refers to factors that encourage uptake of VMMC by males, such as the influence of female partners through acceptance of the procedure, provision of health information about VMMC, etc.
Barriers	Factors that discourage men from undergoing VMMC, such as the individual perception of VMMC being unnatural or unnecessary, negative secondary experiences related to post- VMMC complications such as poor wound healing, etc.
Health system factors	Refers to the healthcare system-related dynamics that influence male client’s response to VMMC. Health system factors encompass all the healthcare-related issues (tangible and non-tangible, which affect uptake of VMMC depending on how they manifest).
Psychosocial factors	These are the social and psychological variables that ultimately influence male client’s response to VMMC. Examples of these factors include societal norms and values regarding VMMC, individual perceptual factors (level of education, individual conceptualisation regarding VMMC etc.).
Family value system	Refers to the family values (traditional, religious, cultural) that a male client is socialised into, which influence individual decision and ultimately the response to VMMC.
Female partners	Refers to the male client’s female counterpart who also influences the individual decision regarding VMMC.
Individual decision	Refers to the male client’s receptiveness to VMMC made on the basis of varying intrinsic and extrinsic factors.

VMMC, voluntary medical male circumcision.

**Concept classification:** The conceptual framework by Dickoff, James and Wiedebach^46^ was used to classify the defined concepts and was also used to develop guidelines to operationalise the model. The framework’s survey list for concept classification consists of the following elements: (1) agent, (2) recipient, (3) context, (4) procedure and (5) dynamics.

#### Step two: Construction of relational statements

The varying categories of concepts were placed in relationships with each other and the respective descriptive relational statements that were developed. This resulted in the formation of a meaningful explanatory structure or model for VMMC as described here.

#### Step three: Description of the model

The guidelines by Chinn and Kramer^44^ for describing theory were used for explaining the following components of the model: the purpose of the model, its assumptions, the context in which it applies, the concepts making up the model, the theoretical definition of concepts and the structure of the model. The researcher subsequently formulated guidelines to operationalise the model. Operationalisation of the model in turn has implications for clinical practice in terms of the quality of VMMC care rendered, the uptake of VMMC and teaching and learning.

#### Step four: Evaluation of the model

For the purpose of this study, the developed model was not evaluated by practical implementation. However, the model was reviewed by an expert in model development in the field of community and public health. The criteria of simplicity, clarity, applicability and importance were used when developing and reviewing the model.

### Ethical considerations

Ethical clearance to conduct the study was obtained prior to data collection from the Biomedical Research Ethics Committee (BREC) of the University of KwaZulu-Natal (BREC reference: BE627/18). Ethical approval was also sought from the KwaZulu-Natal Department of Health research ethics committee. Gatekeeper permission was obtained from the data collection sites prior to the collection of data. Informed consent was obtained from all participants, verbally and in writing before the collection of data.

## Results

### Purpose of the model

This model serves as a framework for healthcare service providers and policymakers to provide awareness of men’s response to VMMC. The knowledge acquired through the model has implications for clinical practice in terms of promoting the uptake of VMMC and the continuous quality improvement of the service delivered. The model aims to have practical applications for teaching and learning for the VMMC training of HCWs at PHC level, in relation to demand creation.

### Assumptions of the model

According to the literature, assumptions are basic principles or statements that are seen as being true without being verified.^44,47^ The assumptions of the model are based on a synthesis of the research findings, which have been interpreted in relation to the literature reviewed and, on the belief, that male clients are physical, psychological, spiritual, emotional and social beings who are in constant interaction with their environment and as such make decisions influenced by their environment. As social beings, socialisation has occurred from infancy and continues to occur daily through values and descriptions depicted by society and other environmental factors such as family norms, customs and beliefs.^48^

The assumptions in this model are as follows:

- The men’s world views are influenced by the *context* (the extrinsic variable) and will determine *influential* (intrinsic variable) and *outcome* variables.
- The decision to undergo VMMC is shaped by a complex interplay of a combination of interrelated factors, namely the extrinsic (external environment) and intrinsic (experiences, understanding and conceptions) factors regarding VMMC. These factors are the key influential variables affecting a male client’s response to VMMC, the outcome.
- Depending on the context in which males find themselves, specific aspects such as psychosocial factors, health system factors, the family value system, female partners or significant others influence decisions made by the male as an individual. Such influences result in specific individual factors that collectively lead to the formation of specific experiences, conceptions and understanding regarding VMMC.
- There are intrinsic (internal) environmental factors that can, in turn, influence how the individual experiences, understands and perceives their external environment.
- The internal and external influences of the individual male are in turn shaped by a complex interplay of interrelated experiences, understanding and conceptions, which collectively act to influence the decision made by a male regarding VMMC.
- Negative individual experiences, understanding and conceptions regarding VMMC act as barriers to uptake and vice versa.
- Demand-generative activities for the uptake of VMMC are more effective when extended to consider the array of contextual factors that influence an individual’s response to VMMC.
- Depending on the environment of the male, the individual family value system may form the foundation upon which experiences, understanding and perceptions are formulated.
- The decision to undergo VMMC is one that cannot be quantified and arises from many external and internal influences, both negative and positive. The negative influences are a potential threat to the long-term health benefits of VMMC.

### The context in which the model applies

This model is applicable to the KZN context and may inform the development of a relevant strategy to facilitate uptake of VMMC particularly in a rural PHC context. The success of such a strategy may be reliant upon a consideration of the array of factors influencing men’s responses to VMMC and community engagement, multisectoral collaboration and the adoption of a health-promotive approach to healthcare.

### Classification of concepts

The survey list by Dickoff et al.^46^ was used to classify concepts as follows: agent, recipient, context, procedure, dynamics and terminus. The *agent* refers to an individual who takes an active role in ensuring the production of a specific desired effect. In this case, the desired effect is an increase in the uptake of VMMC. In the model, the agent refers to the healthcare service provider (nurse, clinical associate or doctor) and the health policymaker.

The *recipient* is the person who attains something. In this case, the recipient receives tailored knowledge and VMMC services. The recipients in this study are uncircumcised males and their primary influencers (female partners, HCWs and medically circumcised males). The uncircumcised males are aged 15 years and older and generally have a higher risk of contracting an HIV infection. The primary influencers have a direct and indirect impact on the decision of a male to undergo VMMC.

The *context* refers to the setting, conditions or situation in which events occur. In this study, the context refers to the PHC setting with high HIV and low male circumcision prevalence.

A *procedure* is a set of specific activities that are required to ensure the realisation of the intended goal. In this study, the procedure refers to the interventions that are needed to achieve the scale-up of VMMC in KZN. Generally, these activities encompass influencing individual male perceptual factors and those factors of primary influence based on their experiences, understanding and conceptions.

The *dynamics* refer to the entities that bring about changes during the process. In this model, the dynamics are the enabling factors that promote the uptake of VMMC. Amongst these are the educative interactions that affect male clients regarding VMMC.

*Terminus* refers to the result to be obtained at the end of the system or process. In this case, it is the improved uptake of VMMC by males aged 15 years and older, the potential being a decreased incidence of HIV.

### Formulation of relational statements

A model refers to a set of interrelated concepts that stipulate the manner in which statements or concepts relate to each other in the formation of the substance of a model. The following are the relational statements proposed between and amongst the concepts identified:

- The term context is used interchangeably with the term external environment and refers to the setting in which an array of influencing factors arise. These factors are as follows: the family value system (including religious, traditional and cultural norms), female partner, psychosocial factors and health system factors, each of which influence and result in specific individual male influencing factors. In a specific context, these factors collectively form experiences, understanding and conceptions regarding VMMC.
- An interrelation of experiences, understanding and conceptions regarding VMMC, results in specific barriers and enablers that affect the response to VMMC.
- An interaction between the enabler and the barrier constructs of the model has a direct effect on the uptake of VMMC by male clients. As the barriers are addressed through a series of interventions such as the provision of tailored messaging, the enabler constructs are promoted. Once the enablers outweigh the barriers, the uptake of VMMC is improved.

### Description of the structure of the model

**FIGURE 2:**
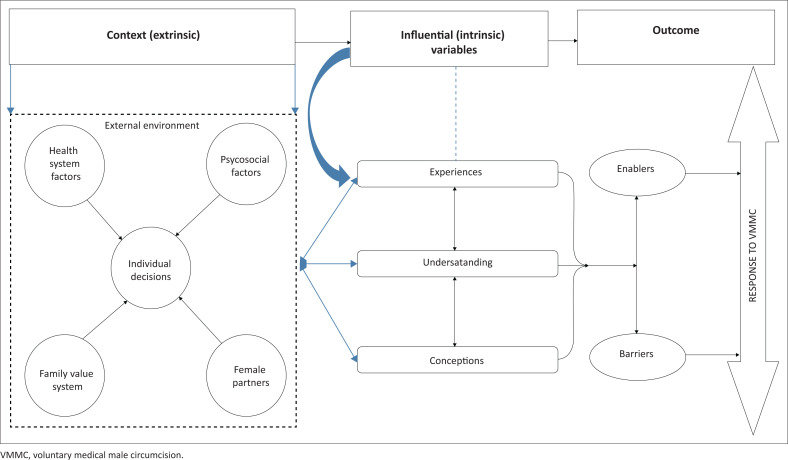
Proposed explanatory model of voluntary medical male circumcision in KwaZulu-Natal, South Africa.

**FIGURE 3:**
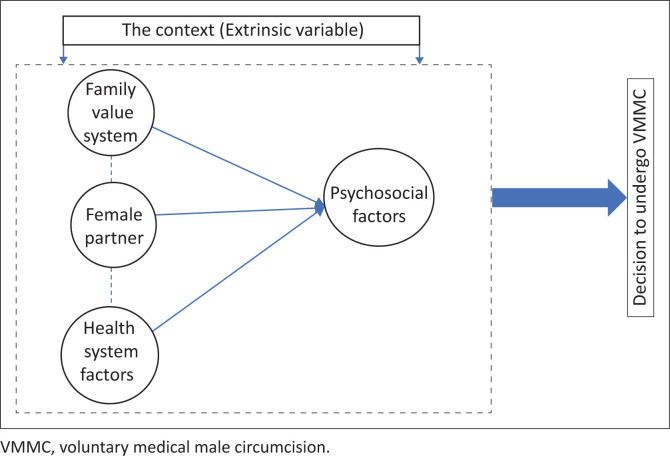
Interaction of extrinsic factors.

The diagrammatic illustration is a structural representation of the overarching conceptual relations within the model. Chin and Kramer^49^ have proposed that concepts be put diagrammatically to depict the core concepts. In developing the model, the experiences, understanding and conceptions of participants were central as they provided information on the barriers to and drivers of VMMC. In so doing, they provided data on how VMMC is understood in the KZN context, the result being a guide to possibly inform interventions for uptake. See Figure 2 for diagrammatic representation of the proposed explanatory model:

- The square structure made up of broken lines represents the external environment or context (extrinsic variable), which harbours the psychosocial factors, health system factors, family value systems and female partners. Central to these factors is the individual decision, which is influenced by the above-mentioned external factors. See Figure 3 outlining the interaction between the extrinsic variables in the context. The three arrows pointing towards the influential variables are indicative of the influence of the extrinsic variable, which influences the intrinsic variables, resulting in specific experiences, understanding and conceptions regarding VMMC. Depending on how these unfold, they result in barriers and enabling factors that affect the male client’s response to VMMC (i.e. they influence the outcome variable). The context or environment is where the socialisation of males occurs, this is the process whereby awareness of norms, values, precepts, skills and habits are developed as shared by family and the society in which they live.^50^ According to the social cognitive theory, social, cognitive and cultural processes are always at work in human beings. Through socialisation, men are in constant interaction with the external environment and as such make certain decisions as influenced by this constant interaction.^51^
- In relation to the uptake of VMMC, acceptance of the procedure is a dynamic process in which personal factors, environmental factors and human behaviours exert and influence each other in the process of decision making, resulting in a specific response to VMMC. Central to this dynamic process is the individual decision, which comprises amongst other aspects of the core individual who is a human being. According to the theory of reasoned action, human being makes systematic use of the information available to them in order to inform decisions related to health behaviour, VMMC being one of such health behaviours.^52^ The information for making such decisions is obtained from the external environment and arises from psychosocial factors, health system factors, the influence of the family value system (religious, traditional and cultural norms) and the female partner.
- The barriers and enabling factors are depicted in the oval structures, and their influence on the response to VMMC is shown by the arrows arising from each oval structure, which, respectively, point towards the main two-way arrow depicting the response to VMMC.

## Evaluation of the model

The model was evaluated by expert researchers in the fields of public health, PHC and HIV care. The criteria of relevance, applicability, clarity, user friendliness and originality of work were used to evaluate the model. The key terminology and relational statements were clearly explained, and a detailed description of the structure of the model was provided. A simple and comprehensive diagrammatic illustration was subsequently provided. The model was found to be relevant because it was designed using data that were generated through an in-depth qualitative phenomenographic study. The constructs of the model, in particular the influential variables and enabling variables, show a clear link between the context and target audience. The relational statements provided describe how factors within individual male experiences may also influence the uptake of VMMC. This frames the contextual and intrinsic factors that HCWs need to consider. Overall, the model was found to be clear and simple because the processes within the diagrammatic structure could be easily followed. Moreover, it was found to be original because it was developed from the qualitative findings of a phenomenographic enquiry into the qualitative differences in primary health stakeholders’ experiences, understanding and conceptions of VMMC in KZN, South Africa. The rural context in which the study was conducted also adds to the overall uniqueness of the findings and subsequently the model developed therefrom.

## Conclusion

The aim of this study was to develop and describe an explanatory model of VMMC in KZN, South Africa. The area of VMMC is continuously evolving and very little is known, particularly regarding the experiences, understanding and perceptions of males and how these may inform and direct health service providers in terms of aligning services and interventions, which are contextually relevant, especially in KZN, South Africa. The model describes how contextual, extrinsic and intrinsic variables interact to determine an individual male’s response to VMMC, thus providing a guide to HCWs on care, practice and policy interventions with specific relevance to the rural PHC context.
